# Autophagy upregulation as a possible mechanism of arsenic induced diabetes

**DOI:** 10.1038/s41598-018-30439-0

**Published:** 2018-08-10

**Authors:** Marzieh Zeinvand-Lorestani, Heibatullah Kalantari, Mohammad Javad Khodayar, Ali Teimoori, Najmaldin Saki, Akram Ahangarpour, Fakher Rahim, Soheila Alboghobeish

**Affiliations:** 1 Toxicology Research Center, Ahvaz Jundishapur University of Medical Sciences, Ahvaz, Iran; 2 Department of Toxicology, School of Pharmacy, Ahvaz Jundishapur University of Medical Sciences, Ahvaz, Iran; 3 Department of Virology, School of Medicine, Ahvaz Jundishapur University of Medical Sciences, Ahvaz, Iran; 4 Health Research Institute, Research Center of Thalassemia and Hemoglobinopathy, Ahvaz Jundishapur University of Medical Sciences, Ahvaz, Iran; 5 Health Research Institute, Diabetes Research Center, Department of Physiology, Ahvaz Jundishapur University of Medical Sciences, Ahvaz, Iran; 6 Department of Pharmacology, School of Pharmacy, Ahvaz Jundishapur University of Medical Sciences, Ahvaz, Iran

## Abstract

The key features of type 2 diabetes mellitus (T2DM) caused by high fat diet (HFD) in combination with arsenic (As) exposure (pronounced glucose intolerance despite a significant decrease in insulin resistance) are different from those expected for T2DM. Autophagy has been considered as a possible link between insulin resistance and obesity. Therefore in this study, we utilized autophagy gene expression profiling via real-time RT-PCR array analysis in livers of NMRI mice exposed to an environmentally relevant and minimally cytotoxic concentration of arsenite (50 ppm) in drinking water while being fed with a HFD for 20 weeks. Out of 84 genes associated with autophagy under study, 21 genes were related to autophagy machinery components of which 13 genes were downregulated when HDF diet was applied. In this study, for the first time, it was shown that the exposure to arsenic in the livers of mice chronically fed with HFD along with increased oxidative stress resulted in the restoration of autophagy [upregulation of genes involved in the early phase of phagophore formation, phagophore expansion and autophagosome-lysosome linkage stages]. Considering the role of arsenic in the induction of autophagy; it can be argued that reduced insulin resistance in HFD − As induced diabetes may be mediated by autophagy upregulation.

## Introduction

Type 2 diabetes mellitus is characterized by impaired insulin secretion, insulin resistance, overproduction of glucose by the liver, and an abnormal fat metabolism. Obesity, especially of visceral or central type, is common in type 2 diabetes (up to 80% of diabetics are obese)^1^. Unquestionably, factors such as genetics, diet, and lifestyle are not the only factors increasing the prevalence of diabetes in recent years, and environmental pollution is likely to be a contributing factor^2,3^. Among the environmental pollutants, arsenic has attracted more attention. Epidemiological studies conducted in Bangladesh, Taiwan, Mexico, and the United States indicate a significant increase in the incidence of diabetes due to the presence of this compound in potable water^4–7^.

Recent studies have shown that the key characteristics of diabetes in HFD mice treated with inorganic form of arsenic are different from those expected from type 2 diabetes (pronounced glucose intolerance despite a significant decrease in insulin resistance)^8–12^. Autophagy is the main pathway for clearing the cells from senescent proteins or other damaged intracellular structures with impaired functions, and the dysregulation of this process is involved in several diseases and aging^13,14^. In recent years, there has been a better understanding of the relationship between autophagy and various types of metabolic stresses. There are several types of autophagy in the cells, which are often simultaneously active; however, the type of degradation performed by them differs according to the type of cell and cellular conditions^15^. Macroautophagy, microautophagy, and chaperone-mediated autophagy are three known forms of autophagy, among which macroautophagy has a dual role in biogenesis and degradation of lipid droplets^16–18^. Previous studies show autophagy involvement in cellular events and liver hemostasis. It has previously been shown that hormones and nutrition regulate the activity of this catabolic process in the liver and other organs; however, in recent studies focusing on this pathway, it has been shown that degradation through autophagy is not only valuable for maintaining hemostasis in the liver, but it also affects other liver events, such as metabolic disorders, lipotoxicity, proteinotoxicity, infection, carcinogenesis, and pathogenesis of common liver disease^19–21^. Two of the most important functions of autophagy in the liver include cellular energetic balance and cellular quality control. Cellular energy balance is achieved through the breakdown of intracellular macro-molecular reserves, organelle turnover (especially in the mitochondria), and the control of key enzymes regulating cell metabolism^19–21^. Lysosomal defective amino acids can either be used to make new proteins, or enter into the Kerbs cycle to make ATP and gluconeogenesis, and then be consumed^22–24^. Activation of macroautophagy in the liver increases glucagon levels and decreases insulin levels, both of which are autophagy inhibitors^20,25–27^. Macroautophagy in liver cells also has another cargo called lipids. A process by which fatty particles, cytosolic triglyceride reservoirs, and cholesterol esters accumulate into autophagosomes and are broken up by lysosomal lipase is called macrolipophagy^18,28^. During dietary lipid overload, macrolipophagy inhibits fat accumulation in the liver^20^. Autophagy can also act to maintain the balance survival of energy in the liver through the glycogen catabolism, which is one of the important sources of energy storage in the liver^29–31^. This process known as glycophagy, is carried out through the participation of the Atg protein called GABARAPL1 (γ-aminobutyric acid receptor associated protein-like 1) and the cargo-recognizing receptor called stbd1 (starch-binding domain-containing protein 1)^20,31^. Recent studies have shown that the energetic imbalance due to the specific deletion of the essential autophagy genes in the liver leads to a defect in the ability to regulate the body’s homeostasis during exposure to lipolysis, proteolysis, and other forms of nutritional stress^20^. An energy imbalance in animals with blocked autophagy will be intensified with a defect in mitochondrial function and this destructive cycle is due to the inaccurate turnover of this organelle due to a defect in autophagy (mitophagy)^20,32^. Chronic lipogenic stimuli or over nutrition in long-term leads to the disruption of macroautophagy. Studies have shown that down regulation of autophagy can contribute to complications of obesity and diabetes, which appear as endoplasmic reticulum stress (ER stress) and insulin resistance^13,33^. The mitochondria are the primary sources of reactive oxygen species (ROS) production, and ROS is capable of activating autophagy through the inhibition of mammalian target of rapamycin complex 1 (mTORC1) and p53^34–38^. In our previous studies, we found that hepatotoxicity, oxidative stress, and liver mitochondrial damage were involved in HFD − As diabetes^39^.

There have been few studies concerning arsenic-induced changes on autophagy pathway in liver cells^40–43^. In a study, it was shown that arsenic could induce autophagy via a ROS-dependent pathway^44^. Therefore, the aim of this study was to specify the involvement of autophagy in diabetogenic effects of chronic exposure to inorganic arsenic and HFD. In particular, the hypothesis is posed that considering the role of arsenic and ROS in the induction of autophagy, it can be argued that reduced insulin resistance in HFD − As diabetes is mediated by autophagy upregulation. The findings from this study can increase our knowledge for the prevention and treatment of arsenic-dependent T2DM.

## Results

### Liver distribution of arsenic

Arsenic exposure led to the accumulation of this agent in the livers of LFD + As and HFD + As treated mice (p < 0.001) (Table 1).

**Table 1:** Liver distribution of arsenic FBG, FSI, HOMA.IR, HOMA.β and oxidative stress markers in the control (LFD or HFD) and arsenic treated mice (LFD50 or HFD50).

Variable	LFD	LFD50	HFD	HFD50
Liver distribution of Arsenic (ng/g)	12.73 ± 3.520	310.6 ± 29.72^***^	9.225 ± 1.995	276.7 ± 17.28^###^
FBG (mg/dL)	101.8 ± 7.314	139.5 ± 13.85	207.7 ± 15.92^***^	114.5 ± 10.98^###^
FSI (ng/L)	0.3099 ± 0.06996	0.1607 ± 0.008793^**^	0.1698 ± 0.02203^**^	0.09067 ± 0.03320^#^
HOMA.IR(arbitrary unit)	6.100 ± 0.6671	4.790 ± 0.2883	9.705 ± 0.8305^*^	2.525 ± 0.7973^###^
HOMA.β(arbitrary unit)	186.0 ± 35.33	117.0 ± 36.68^*^	41.25 ± 9.232^**^	20.75 ± 5.588^#†^
ROS (FIU/g)	160.2 ± 0.28	221.3 ± 17.1^**^	192.5 ± 10.2	296.8 ± 26.22^###^
MDA (nmol/g)	10.53 ± 2.804	28.62 ± 3.308^***^	30.62 ± 2.085^***^	52.06 ± 1.953^***###^

Values represented as mean ± SE (n = 12). *Significantly different from LFD, ^#^Significantly different from HFD. ^†^Significantly different from LFD + As 50 ppm. *^,#^ and $ p < 0.05, ** and ^##^p < 0.01, *** and ^###^p < 0.001.

### Serum glucose and insulin

FBG and FSI were administered to evaluate glucose homeostasis and insulin secretion in response to glucose challenge and insulin resistance, respectively. After 20 weeks of LFD consumption, the control LFD mice showed an average FBG level of 101.83 mg/dL. HFD feeding for 20 weeks resulted in a significant increase of FBG (207.7 mg/dL) in control HFD mice (*p* < 0.001). Furthermore, lower FBG levels were detected in HFD mice exposed to 50 ppm of arsenic (114.5 mg/dL) in comparison with the control HFD group (*p* < 0.001).

In general, the mice in the HFD groups showed lower FSI levels compared to the LFD group (*p* < 0.01). Compared to the control HFD mice, FSI value in HFD + As exposure group was decreased (*p* < 0.05). Also, LFD + As (50 ppm) revealed a significant reduction in FSI when compared to the control LFD group (*p* < 0.01).

The averages of HOMA-IR values were consistently higher in the control HFD than LFD group (*p* < 0.05). As exposure did not change HOMA-IR in LFD-fed mice, but it decreased this index in the HFD group (*p* < 0.001). The value of HOMA-β was significantly reduced in the HFD control group compared to the LFD control mice (*p* < 0.05). Further exposure to As (50 ppm) significantly decreased the HOMA-β values in LFD- and HFD-fed mice compared to their control groups (*p* < 0.01 and *p* < 0.05, respectively) (Table 1).

### Hepatic oxidative stress

As shown in Table 1, HFD induced a rise in ROS formation in the liver. Exposure to As (50 ppm) increased this variable in LFD-fed mice (p < 0.01) compared to its control. Also, compared to the control HFD group, overproduction of ROS has occurred in HFD + As (50 ppm) treated mice (p < 0.001). The results of lipid peroxidation revealed that liver MDA level was significantly higher in the control HFD mice compared to LFD group (p < 0.001). In addition, As (50 ppm) exposure significantly increased this variable in mice fed with LFD and HFD (p < 0.001) when compared to their controls.

### Transcriptomic analysis of autophagy gene expression in HFD, LFD − As, and HFD − As treated groups

**Figure 1:**
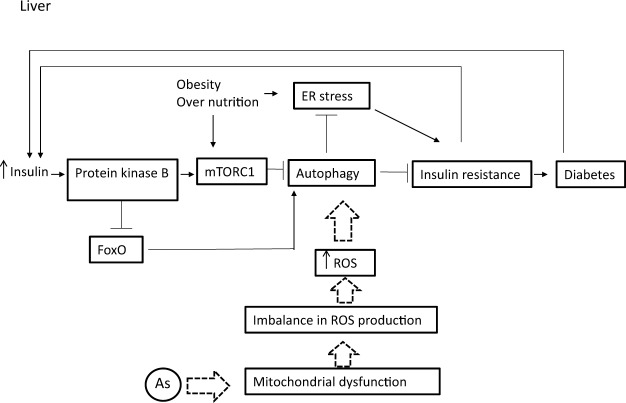
Involvement of autophagy in diabetogenic effects of chronic exposure with inorganic arsenic and the use of HFD simultaneously. Macroautophagy is inhibited by insulin amino acid-mTOR signaling pathway in short- or long-term patterns. Short-term inhibition can be generated through mTORC1 by preventing ULK1 and long-term inhibition is achieved through FoxO transcription factors (subgroup of the Forkhead family of transcription factors) controlling the transcription of genes involved in autophagy. FoxO is phosphorylated and inhibited by insulin-induced action of PKB (Protein kinase B). Continuous intake of energy and nutritional stress inhibit autophagy that causes metabolic stress, insulin resistance, and type 2 diabetes. In particular, the hypothesis is posed that considering the role of arsenic in the induction of autophagy, it can be argued that reduced insulin resistance in HFD − As diabetes is mediated by autophagy upregulation.

**Figure 2:**
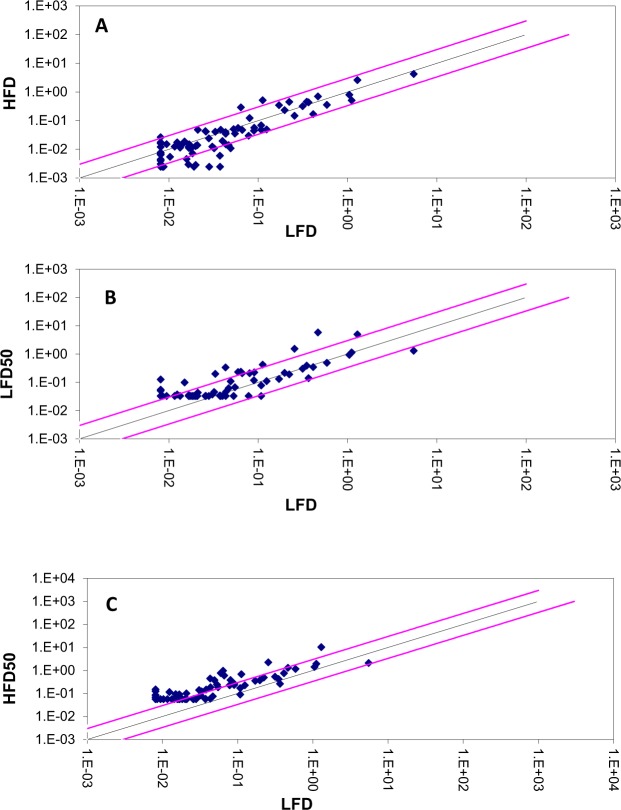
Presents change in the expression of autophagy genes. The Scatter Plot graphs show the expression level (${2}^{-{\rm{\Delta }}\text{CT}}$) of each gene in the control sample versus the test sample. The black line indicates fold changes (${2}^{-{\rm{\Delta }}{\rm{\Delta }}\text{CT}}$) of 1. The pink lines indicate the desired fold-change in gene expression threshold. (**A**) Changes in expression of autophagy genes in HFD treated groups. (**B**) Changes in expression of autophagy genes in LFD − As treated groups. (**C**) Changes in expression of autophagy genes in HFD − As treated groups.

To investigate the hypothesis outlined in this study (Fig. 1), the expressions of autophagy pathway and genes in LFD − As, HFD and HFD − As treatments were studied by RT2 Profiler PCR Arrays-Mouse Autophagy. HFD significantly reduced the expressions of several autophagy-related indicators in the livers of mice (Fig. 2A). In contrast, majority of these indicators were increased in the liver tissues of LFD50 mice compared to the control group (Fig. 2B). In confirmation of the hypothesis set forth in this study, the livers of mice fed with HFD that were exposed to arsenic showed a restoration in the expression of genes involved in autophagy (Fig. 2C). The results are displayed as a “Scatter Plot” (Fig. 2A–C). The findings of this study show a significant change of markers that play a critical role in autophagy machinery components in CMA and macroautophagy, which will be further discussed in more detail.

### Macro autophagy machinery components

The genes and proteins regulating autophagy (as effectors and modulators) are different in the three types of autophagy, including macroautophagy, microautophagy, and chaperone-mediated autophagy^16^. Microautophagy regulates the entry of cytosolic cargo-proteins and organelles through the invagination of lysosomal membrane, and the molecular components involved in this type of autophagy have not been elucidated^17,45^. As shown in Table 2, the findings of this study show a significant change of markers that play a role in the pathway and structure of autophagy in macroautophagy and CMA. Out of the 84 genes associated with autophagy under study, 21 genes were related to autophagy machinery components of which 13 genes were downregulated when HDF diet was applied. Remarkably, the exposure to arsenic in this study led to the upregulation of all the examined genes involved in macroautophagy machinery components as well as chaperone-mediated autophagy (CMA) when the HFD was received (Table 2).

**Table 2:** Autophagy gene expression profiling regulated with arsenic exposure while fed with HFD or LFD with 1 fold alteration (up- or downregulated) by PCR array.

Symbol	AVG ΔC_t_ (Ct(GOI) − Ave Ct (HKG))				${2}^{-{\boldsymbol{\Delta }}{\bf{C}}{\bf{T}}}$ ^*^				Fold Change**			Fold Up- or Down-Regulation***		
	LFD50	HFD	HFD50	LFD	LFD50	HFD	HFD50	LFD	LFD50/LFD	HFD/ LFD	HFD50/ LFD	LFD50/ LFD	HFD/ LFD	HFD50/LFD
Atg12	4.92	6.06	4.15	6.73	3.3E − 02	1.5E − 02	5.6E − 02	9.5E − 03	**3.49**	1.59	**5.96**	**3.49**	1.59	**5.96**
Atg16l1	2.25	4.41	0.76	3.92	2.1E − 01	4.7E − 02	5.9E − 01	6.6E − 02	**3.17**	0.71	**8.91**	**3.17**	−1.41	**8.91**
Atg16l2	4.92	8.68	4.10	4.74	3.3E − 02	2.4E − 03	5.8E − 02	3.8E − 02	0.88	*0.07*	1.55	−1.14	*−15.37*	1.55
Atg3	4.92	5.08	2.15	3.68	3.3E − 02	3.0E − 02	2.3E − 01	7.8E − 02	*0.42*	*0.38*	**2.88**	*−2.37*	*−2.64*	**2.88**
Atg4b	4.18	5.26	3.02	6.95	5.5E − 02	2.6E − 02	1.2E − 01	8.1E − 03	**6.79**	**3.22**	**15.19**	**6.79**	**3.22**	**15.19**
Atg4c	3.20	6.53	1.41	4.35	1.1E − 01	1.1E − 02	3.8E − 01	4.9E − 02	**2.21**	*0.22*	**7.65**	**2.21**	*−4.54*	**7.65**
Atg5	3.08	4.50	2.11	3.48	1.2E − 01	4.4E − 02	2.3E − 01	9.0E − 02	1.31	*0.49*	**2.58**	1.31	*−2.03*	**2.58**
Atg7	4.15	7.79	4.15	5.99	5.6E − 02	4.5E − 03	5.6E − 02	1.6E − 02	**3.57**	*0.29*	**3.57**	**3.57**	*−3.49*	**3.57**
Atg9a	4.92	8.68	4.15	6.88	3.3E − 02	2.4E − 03	5.6E − 02	8.5E − 03	**3.87**	*0.29*	**6.61**	**3.87**	*−3.49*	**6.61**
Becn1	4.92	5.96	4.15	6.95	3.3E − 02	1.6E − 02	5.6E − 02	8.1E − 03	**4.06**	1.98	**6.94**	**4.06**	1.98	**6.94**
Gabarap	1.24	0.96	0.56	3.16	4.2E − 01	5.1E − 01	6.8E − 01	1.1E − 01	**3.76**	**4.59**	**6.04**	**3.76**	**4.59**	**6.04**
Gabarapl1	−0.20	0.98	−0.95	−0.16	1.1E + 00	5.1E − 01	1.9E + 00	1.1E + 00	1.03	*0.46*	1.74	1.03	*−2.19*	1.74
Gabarapl2	3.90	4.84	2.43	4.19	6.7E − 02	3.5E − 02	1.9E − 01	5.5E − 02	1.22	0.64	**3.38**	1.22	−1.57	**3.38**
Hdac6	4.92	6.36	4.15	5.91	3.3E − 02	1.2E − 02	5.6E − 02	1.7E − 02	1.98	0.73	**3.38**	1.98	−1.37	**3.38**
Hsp90aa1	2.07	1.80	0.05	3.97	2.4E − 01	2.9E − 01	9.7E − 01	6.4E − 02	**3.71**	**4.49**	**15.08**	**3.71**	**4.49**	**15.08**
Hspa8	−2.31	−1.37	−3.34	−0.38	5.0E + 00	2.6E + 00	1.0E + 01	1.3E + 00	**3.82**	2.00	**7.81**	**3.82**	2.00	**7.81**
Lamp1	2.92	1.55	1.52	2.55	1.3E − 01	3.4E − 01	3.5E − 01	1.7E − 01	0.77	2.00	**2.03**	−1.30	2.00	**2.03**
Map1lc3a	4.15	5.98	4.15	6.12	5.6E − 02	1.6E − 02	5.6E − 02	1.4E − 02	**3.90**	1.10	**3.90**	**3.90**	1.10	**3.90**
Npc1	4.92	4.86	4.08	4.53	3.3E − 02	3.4E − 02	5.9E − 02	4.3E − 02	0.76	0.79	1.36	−1.32	−1.26	1.36
Ulk1	4.86	7.39	4.15	4.75	3.4E − 02	6.0E − 03	5.6E − 02	3.7E − 02	0.92	*0.16*	1.51	−1.08	*−6.24*	1.51
Wipi1	4.75	6.35	2.84	5.03	3.7E − 02	1.2E − 02	1.4E − 01	3.1E − 02	1.21	*0.40*	**4.55**	1.21	*−2.50*	**4.55**

*${2}^{-{\rm{\Delta }}\text{CT}}$ (normalized gene expression) is the expression levels of gene of interest (GOI) divided the expression levels of HKG genes.

$$\frac{{2}^{-{\rm{CT}}({\rm{GOI}})}}{{2}^{-{\rm{CT}}({\rm{HKG}})}}={2}^{-[{\rm{CT}}({\rm{GOI}})-{\rm{CT}}({\rm{HKG}})]}={2}^{-{\rm{\Delta }}\text{CT}}$$.

**Fold-Change (${2}^{-{\rm{\Delta }}{\rm{\Delta }}\text{CT}}$) is the normalized gene expression (${2}^{-{\rm{\Delta }}\text{CT}}$) in the Test Sample divided the normalized gene expression (${2}^{-{\rm{\Delta }}\text{CT}}$) in the Control Sample.

$\frac{{2}^{-{\rm{\Delta }}\text{CT}({\rm{expt}})}}{{2}^{-{\rm{\Delta }}\text{CT}({\rm{ctrl}})}}={2}^{-{\rm{\Delta }}{\rm{\Delta }}\text{CT}}$ where ΔΔCT is equal to ΔCT (expt) − ΔCT (ctrl).

***Fold-Regulation represents fold-change results in a biologically meaningful way.

Fold-change values greater than one indicate a positive- or an up-regulation, and the fold-regulation is equal to the fold-change. Fold-change values less than one indicate a negative or down-regulation, and the fold-regulation is the negative inverse of the fold-change. Fold-change and fold-regulation values greater than 2 are indicated in bold; fold-change values less than 0.5 and fold-regulation values less than −2 are indicated in italic.

### KEGG pathway of macroautophagy in LFD − As, HFD and HFD − As treated groups

**Figure 3:**
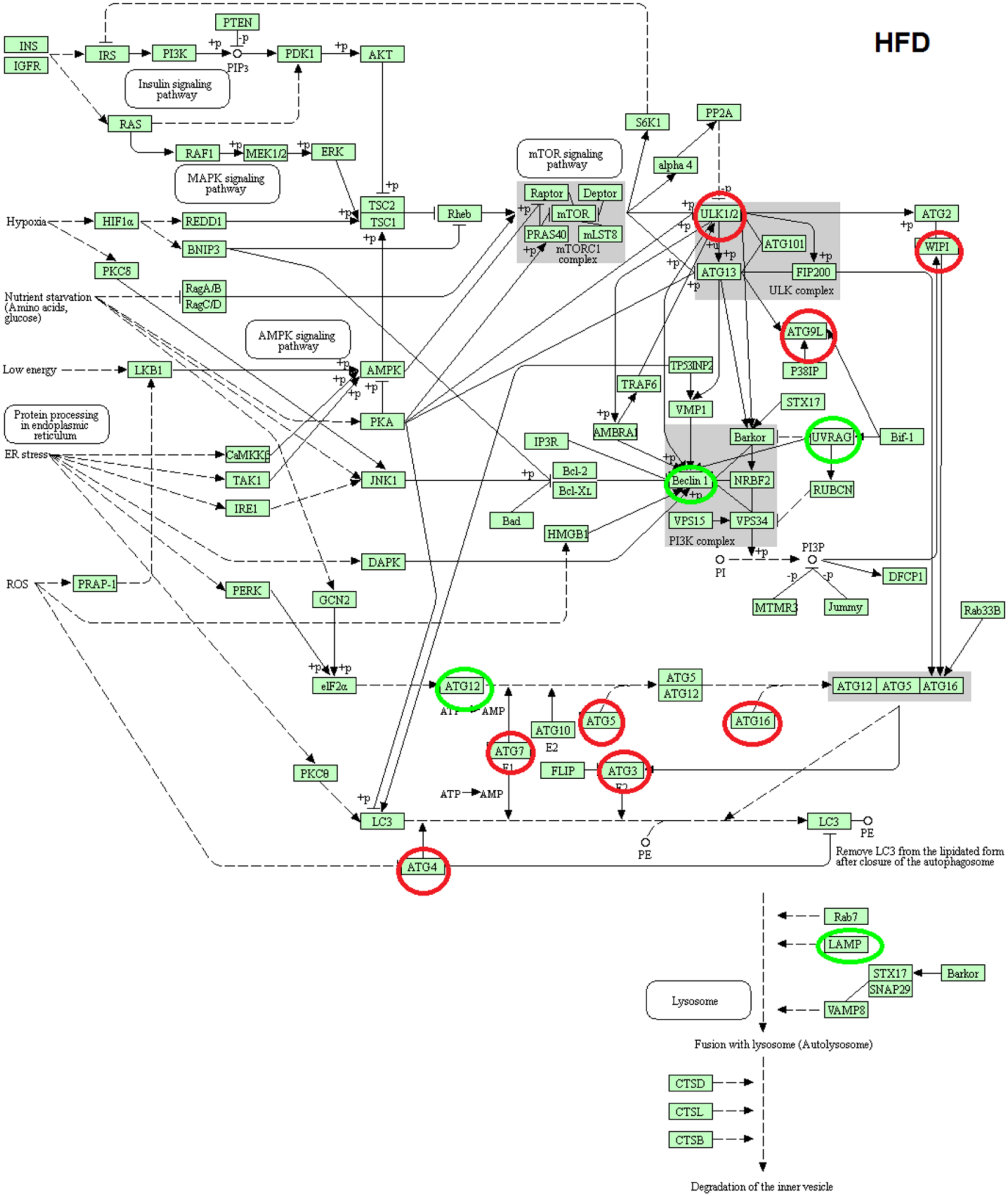
KEGG pathway of differentially macroautophagy hepatic gene expression and pathways http://www.kegg.jp/kegg/kegg1.html^49,50,53^. Changes in expression of autophagy genes in HFD treated groups (the up regulated genes are indicated by green circles and the down regulated genes are indicated by red circles).

**Figure 4:**
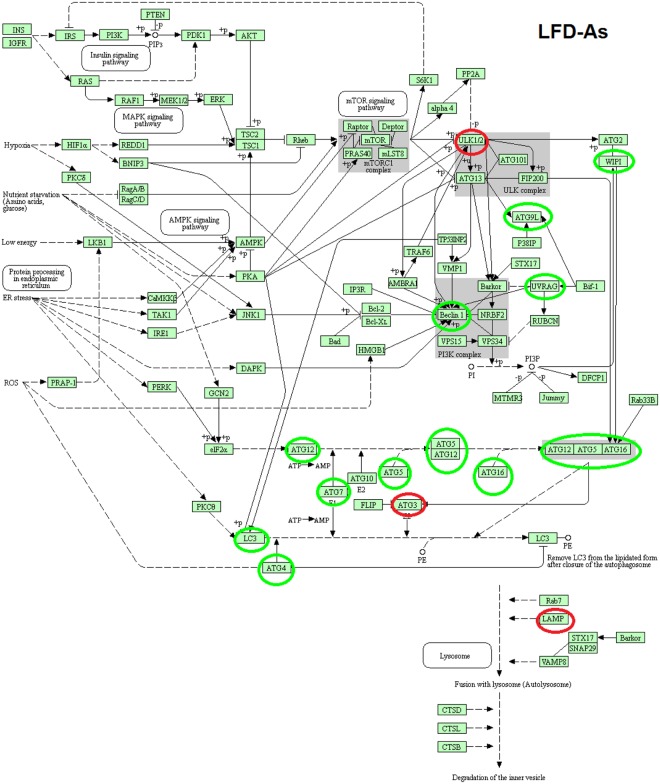
KEGG pathway of differentially macroautophagy hepatic gene expression and pathways http://www.kegg.jp/kegg/kegg1.html^49,50,53^. Changes in expression of autophagy genes in LFD − As treated groups (the up regulated genes are indicated by green circles and the down regulated genes are indicated by red circles).

**Figure 5:**
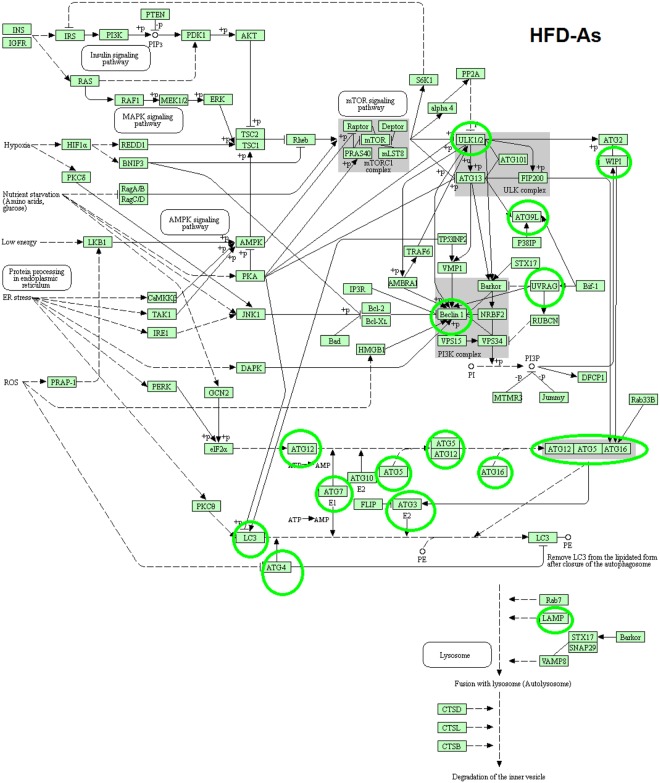
KEGG pathway of differentially macroautophagy hepatic gene expression and pathways http://www.kegg.jp/kegg/kegg1.html^49,50,53^. Changes in expression of autophagy genes in HFD − As treated groups (the up regulated genes are indicated by green circles).

As shown in KEGG pathway (Figs 3, 4 and 5), the macro autophagic pathway involves several specific steps including vacuole formation, ubiquitination, vacuole targeting and autophagosome-lysosome linkage^46–50^. The alteration (upregulation/downregulation) in markers associated with autophagosome formation in the livers of LFD − As, HFD and HFD − As treated groups shows the potential involvement of macroautophagy in the new face of type 2 diabetes.

### Vacuole Formation, Induction and nucleation

**Figure 6:**
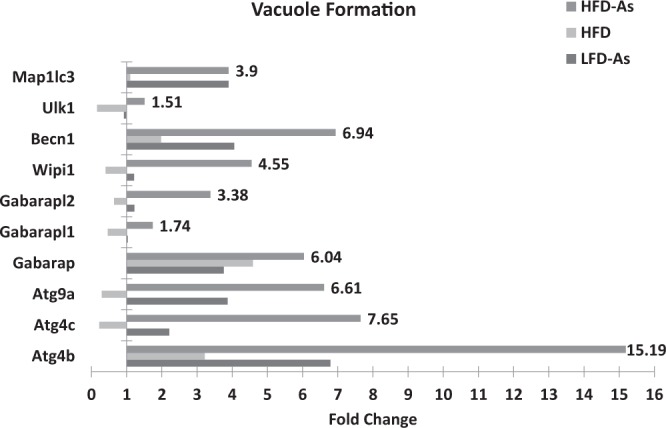
Presents the gene expression profiling of autophagy induction, nucleation and vacuole formation regulated with arsenic exposure while fed with HFD or LFD with 1 fold alteration by PCR array.

The Autophagy-related genes (*ATG*) play an important role in the formation of autophagosomes and the regulation of macroautophagy^51,52^. As shown in Fig. 6, there was a reduction in the expression of most of the genes (Becn1 (BECN), Ulk1 (the Unc-51 like kinase 1 (*C. elegans*)), Atg4c, Atg9a, Gabarapl1, Gabarapl2, Map1lc3b, and Wipi1) involved in the early phase of phagophore formation in livers of HFD mice in comparison with the control group. A nearly contradictory result was observed regarding the majority of genes implicated in this phase in LFD group exposed to arsenic. Additionally, arsenic was able to restore ATG genes expression in the obese mouse liver tissue. Arsenic has induced the expression of Beclin 1, (6.94 fold change), and ULK1 (1.51 fold change) in the HFD50 group. Beclin 1 and ULK1, along with other key subunits, cooperate in the formation of the phosphatidylinositol-3-kinase (PI3K) complex necessary for nucleation and phagophore membrane formation^53^.

### Ubiquitination and phagophore expansion

Elongation of pre-autophagosomal structure requires two ubiquitin-like systems, including Atg12-Atg5-Atg16L (Atg16-like protein) and LC3 conjugation system^54,55^. The microarray analysis indicated that Atg12, Atg5, and Atg16L could show upregulation during exposure to arsenic in the hepatic cells of mice receiving HFD (5.96, 2.58, 8.91 fold change). The ubiquitin-like Atg12 protein is covalently conjugated to the Atg5 protein through the enzymatic activity of Atg7 and Atg10. The conjugated Atg12-Atg5 can non-covalently bind with the Atg16L, and this tetramer (Atg12-Atg5-Atg16L) acts as an e3-like ubiquitin ligase contributing to the development of autophagosomal membrane^51,52,55^. In the second conjugation reaction, ubiquitin-like LC3 is conjugated to phosphatidylethanolamine (PE) by Atg7 and Atg3, and LC3II is thus developed. LC3-II helps the autophagosome membrane to form autophagosome in the elongation phase^56,57^. In the livers of mice receiving HFD, change in the expression of LC3 was roughly at the baseline level (fold change 1.1). This change shows the increasing expression of HFD50 (fold change 3.9) in the livers of mice receiving arsenic. In addition, although decreased expression of Atg3 was observed in the LFD50 group (fold change 0.42), increased expression of Atg3 (fold change 2.88) and Atg7 (fold change 3.58) was seen in mouse liver tissue in the HFD50 group.

**Figure 7:**
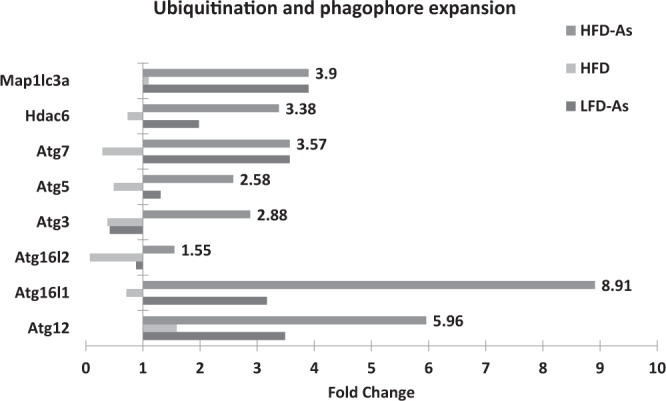
Presents the gene expression profiling of ubiquitination and phagophore expansion regulated with arsenic exposure while fed with HFD or LFD with 1 fold alteration by PCR array.

HDAC6 (Histone deacetylase 6) is critical for ubiquitin-selective quality control of autophagy and autophagosome maturation^58^. High-fat diet resulted in HDAC6 downregulation (fold change 0.73), and arsenic exposure led to its overexpression in LFD50 (fold change of 1.98) and HFD50 (fold change 3.38) groups (Fig. 7).

### Vacuole targeting and autophagosome-lysosome Linkage

**Figure 8:**
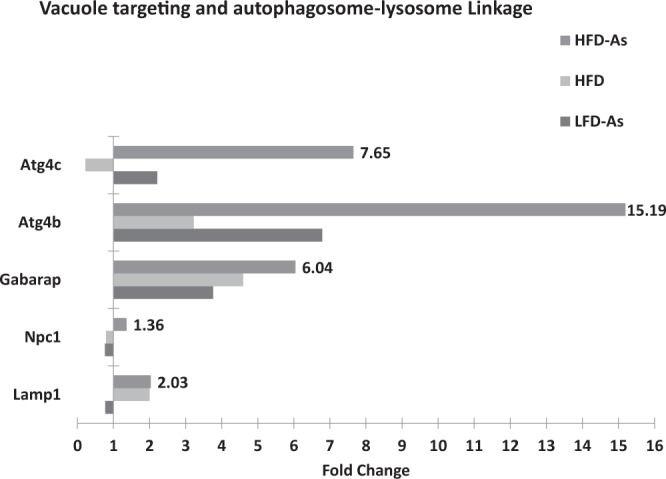
Presents the gene expression profiling of targeting vacuole and autophagosome-lysosome linkage regulated with arsenic exposure while fed with HFD or LFD with 1 fold alteration by PCR array.

After the completion of autophagosome formation, this structure is targeted by tethers/docks and then merged with lysosomal vacuole, which leads to the release of the contents of autophagosome vesicle (autophagic body) into the inner space of lysosomal vacuole^59^. As soon as the autophagic body is removed by lysosome, its contents are degraded by lysosomal lipases and hydrolases. The lysosomal efflux transporters regulate the release of the products (amino acids, fatty acids, and nucleosides) and their restoration into the cytosol^60^. Different proteins are involved in the events associated with this fusion process, some of which have been investigated in this study. In the LFD50 group, there was a reduction in the expression of Npc1, the Niemann Pick type C1, (fold change 0.76) and Lamp1, the Lysosomal-associated membrane protein 1 (fold change 0.77) genes. In the HFD group, reduction (fold change 0.79) of Npc1 expression was observed. There was a significant increase in the expressions of all autophagic indicators examined in the targeting vacuole and autophagosome-lysosome linkage stages in the livers of HFD-fed mice simultaneously receiving arsenic, which was reported as the upregulation of Gabarap, the Gamma-aminobutyric acid (GABA) A receptor-associated protein-like (fold change 6.04), Atg4b (fold change 15.19), Atg4c (fold change 7.65), Lamp1 (fold change 2.03), and Npc1 (fold change 1.36) (Fig. 8).

### Chaperone-mediated autophagy

CMA is a specific cellular pathway that selectively transfers cytosolic protein assemblies into lysosomes to be degraded. In this process, no formation of vesicle or change in the lysosomal membrane is observed^55^. In short, in this study, the expressions of CMA-related genes in HFD, HFD50, and LFD50 groups were significantly higher than in the control group, which showed the highest increase in the HFD50 group. CMA function can be regulated by adjusting the levels of LAMP-2A and Lys-hsc70 in the lysosomes. The heat shock cognate 71 kDa protein (Hsc70), also known as HSPA8, is a member of the heat shock protein 70 family (Hsp70) that plays an important role in the CMA pathway. Hsc70, along with other cochaperones (for example, heat shock protein 90, alpha (cytosolic), class A member 1, Hsc90 protein 90) forms a pentapeptide KFFRQ motif in cytosolic proteins targeted by CMA, forming chaperone/substrate complex^61,62^.

**Figure 9:**
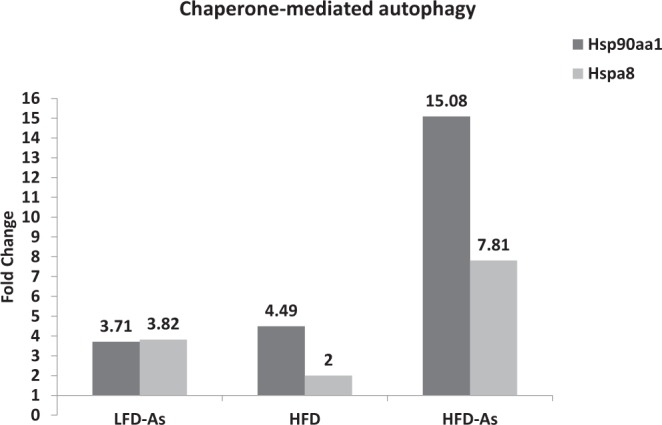
Presents the gene expression profiling of Chaperone-mediated autophagy machinery components regulated with arsenic exposure while fed with HFD or LFD with 1 fold alteration by PCR array.

The high-fat diet led to the upregulation of Hsc70 (fold change 2) and Hsc90 (fold change 4.49). When exposed to arsenic, overexpression of Hsc70 and Hsc90 was seen in LFD50 (fold change 3.82, 3.71) and HFD50 groups (fold change 15.08, 7.81), respectively (Fig. 9).

## Discussion

The data presented here strongly suggest that autophagy is activated in mouse liver and its alterations are likely to be a key component of the cytotoxicity resulting from chronic exposure to arsenic and high-fat diet.

Inorganic arsenic is a global environmental pollutant. Positive relationship of Inorganic arsenic with the incidence of type 2 diabetes mellitus arouses concerns associated with its etiology in diabetes among the general human population^11,63^. Arsenic was increasingly to be blame as a risk factor for type 2 diabetes mellitus. The most widely recognized types of b-cell death in diabetes are apoptosis and necrosis^11,64^. Autophagy was recently recognized in the execution of b-cell death^65^. Alterations in autophagy occur in systemic diseases such as cancer, metabolic dysfunction and in organ-specific pathologies such as neurodegeneration, cardiomyopathies and non-alcoholic fatty liver disease^66–70^. Thus far, numerous studies have been conducted on arsenic-induced changes in autophagy pathways. The results of the studies indicate that the role of arsenic in regulation of autophagy is dependent on the type of cells and cellular stress^63,71–74^. Epidemiological evidence indicates that chronic exposure to arsenic correlates with hepatic injury, such as fibrosis, cirrhosis, and cancer^75–82^.

Liver is an organ in which a high level of metabolic and oxidative stress has been observed due to the presence of mitochondria. Nutrition, energy, and inflammation are associated with obesity-induced stress and lead to insulin resistance^83^. Furthermore, continuous intake of energy and nutritional stress induce inhibitory signaling on autophagy responses, followed by an inadequate function of cellular organelles that cause metabolic decline, insulin resistance, and type 2 diabetes^33,83,84^. Defective autophagy and insulin resistance are highly interrelated and have extensive effects on the metabolic system. In a study conducted by Yang *et al*., ATG suppression caused systemic and hepatic resistance to insulin^33^. In line with studies confirming the role of autophagy in the function and survival of adipocytes, ISLET and liver cells, the data of our study suggest that autophagy is likely to be involved in the apparent mechanism and pathogenesis of type 2 diabetes caused by HFD^13,33,44,83,85^.

In a study by Liu *et al*. in^11^ to determine the effect of arsenic on healthy and diabetic mice, it was shown that although arsenic did not change glucose tolerance in healthy mice, it impaired the function of pancreatic beta cells as well as increased gluconeogenesis and oxidative damage in the liver. In this study and similar studies, it has been proven that insulin resistance does not show the diabetogenic effects of arsenic and that the mechanism leading to manifestations of type 2 diabetes for this toxicant is different from that mentioned for obesity^9,11^. In our previous pilot studies, it was found that hepatotoxicity, oxidative stress, and mitochondrial liver damage are associated with this model of diabetes that is caused by chronic exposure to arsenic and high-fat diet^39^. The expression of beclin-1, which is one of the key components in early phases of autophagy and autophagosome formation, is increased under oxidative stress conditions. It has also been shown that ROS formation is involved in the induction of autophagy through thiol modification of the Cys81 site of Atg4^37,38^. Arsenic is capable of inducing ROS formation. In a study conducted by Zhu *et al*., it was shown that arsenic could cause autophagy via a ROS-dependent pathway^44^.

In our study, for the first time, it was shown that the exposure to arsenic in the livers of mice chronically fed with HFD along with increased oxidative stress resulted in the restoration of autophagy. This study principally shows that not only hepatic autophagy severely rise in HFD − As dietary models of murine obesity for 20 weeks [which was reported as the upregulation of Becn1, Ulk1, Atg4c, Atg9a, Gabarapl1, Gabarapl2, Map1lc3b, and Wipi1 genes (involved in the early phase of phagophore formation and the initiation of autophagy), LC3, Atg12, Atg5, Atg3, Atg7, HDAC6 and Atg16L genes (required for the ubiquitination and phagophore expansion) and Gabarap, Atg4b, Lamp1, and Npc1 (examined in the targeting vacuole and autophagosome-lysosome linkage stages)], but also that this process may underlie the decreased insulin resistance in these models. Considering the role of arsenic in the induction of autophagy, it can be argued that reduced insulin resistance as well as other factors associated with the features of diabetes when using HFD- As may be mediated by autophagy upregulation.

The involvement of autophagy in fat metabolism could be more complex depending on the time of intervention or the age of the experimental animal. Studies have shown that macroautophagy and CMA have a direct relationship and cooperate in macromolecular degradation to maintain survival and homoeostasis^55^. At the time of nutritional stress, the macroautophagic activity increases rapidly, reaching its highest level but decreasing shortly thereafter. If the nutritional stress persists, the activity of CMA pathway can increase and remain active for a long period^55,86,87^. In short, in this study, the expressions of CMA-related genes were significantly higher in HFD, HFD50, and LFD50 groups than in the control group. Macroautophagy defect can lead to the activation of CMA pathway in normal as well as stress conditions^88^. CMA upregulation in response to macroautophagy inhibition improves the clearance of oxidized proteins^89,90^. The compensatory upregulation of each of them is helpful in protecting the cells to withstand a variety of damages and maintain cell survival^88,90^. The therapeutic potentials via targeting the link between macroautophagy and CMA in the treatment of metabolic diseases can be considered in subsequent studies.

Over the past two decades, many findings that emphasize the central role and importance of autophagy in many human diseases have been obtained^91–95^. Different studies on various organisms show the vital role of autophagy in the immune system, metabolic disorders and aging^20,21^. Although research has been ongoing for decades, many of the related events have remained unknown. It seems that regulating the activity of autophagy as an essential catabolic process for the cytoplasmic digestion of compounds by targeting specific regulatory actors in the autophagy process can affect the routine and the occurrence of many diseases^21^. Autophagy has many roles, including extensive regulatory effects on metabolism, an essential role in controlling the quality of organelles and proteins, as well as activating liver defense against pathogens^19–21^. It seems that the complexity of liver autophagy not only originates from its different roles in the physiological and pathological processes, but also it is affected by the various mechanisms involved in its regulation^19–21^. Although it is generally agreed that the activation of autophagy in the liver would have beneficial effects on hepatic physiology, and its protection against liver disease^20,21^. However, the consequences of autophagy which changes due to drug targets or exposure to toxic materials cannot be completely predictable. Therefore, considering that both up-regulation and down-regulation of autophagy have been observed in a variety of diseases, such as metabolic disorders and cancers, it is necessary to identify the key goals of autophagy in a variety of diseases in order to improve the treatment and make pharmaceutical compounds more effective^19,93–95^.

Considering that arsenic activity on liver metabolism may have been related to other autophagy-independent pathways, further studies can examine other mechanisms involved in arsenic function on insulin resistance or other aspects of metabolism.

In general, our study presents important details on the regulation of autophagic responses during homeostasis. Since the more direct evidence can be provided by the Transmission Electron Microscope (TEM) images and LC3 detection we hope that the next researchers will use these methods to complete and improve our study findings. It is possible that small molecules regulating autophagy or the main molecules involved in it could improve the use of this regulatory pathway for therapeutic interventions in type 2 diabetes.

## Materials and Methods

### Animal treatment

Adult male NMRI mice (30–35 g) were obtained from the animal facility of Ahvaz Jundishapur University of Medical Science (AJUMS), which is fully approved by AJUMS animal care guidelines and ethics committee (No. IR.AJUMS.REC.1395.415). All the experiments were performed in accordance with relevant guidelines and regulations. The mice were housed (6 mice per cage) in polycarbonate cages with corncob bedding at 20 ± 4 °C temperature with a 12 h light/12 h dark cycle and 10% humidity.

### Chemicals

Sodium arsenite (99% pure) was purchased from Sigma- Aldrich (St. Louis, MO). A Low-fat diet (LFD, 11% of all calorie supply from fat) and high-fat diet (HFD, 57% of all calorie supply from fat) were obtained from Javaneh Khorasan laboratory, Mashhad, Iran. RNeasy Mini kit, RT2 First Strand Kit, and 96-well RT2 Profiler PCR Arrays-Mouse Autophagy were purchased from Qiagen. Lipid Peroxidation (MDA) Assay Kit and Fluorometric Intracellular Ros Kit were purchased from Sigma-Aldrich (St. Louis, MO). All other commercially available chemicals were of the highest grade.

## Methods

Male mice were divided into four groups of 12 in the form of chronic studies as follows: a low- fat diet control group (LFD) (11% fat, 16% proteins, 72.8% carbohydrate kcal/g) and another, a high-fat diet group (HFD) (58% Fat, 16.4% Protein, 25.5% Carbohydrate, kcal/g). To assess the effect of chronic exposure to arsenic, two other groups received arsenic while consuming HFD (HFD50) and LFD (LFD50). Mouse-specific food was purchased from Javaneh Khorasan Laboratory (Mashhad, Iran), and its arsenic level was determined using atomic absorption by Tehran University Academic Center for Education, Culture and Research. The level of arsenic was approximately 5 and 7 ppb in HFD and LFD, respectively, which was negligible given the 50 ppm applied concentration of arsenic.

Ten times higher concentrations of drinking water arsenic (50 ppm) are needed to achieve liver arsenic concentrations similar to those seen in humans exposed to arsenic in west Bengal. Therefore, in the present study, the livers of mice that drank diH_2_O containing 50 ppm arsenic are used^9^.

Decreased ATG7 expression which is a key indicator of autophagy, occurs following HFD at week 16 and ends at week 22, so the duration of the experimental period was regarded as 20 weeks in this study^33^.

### Serum glucose and insulin analysis

Twenty-four hours after the last experimental day, the overnight fasting animals were anesthetized by ether. Fasting blood glucose was measured by cutting the tail tip and using glucometer (Elegance CT-X10, Convergent Technologies, Germany). Then, blood samples were directly collected by cardiac puncture and centrifuged at 3500 rpm for 20 min. Plasma samples were stored at −70 °C until biochemical assessment was performed. Insulin level measurement was performed by ELISA assay kits (Monobind, USA), and the sensitivity of hormone detection per assay tube was 0.182 µIU/mL. Then, the serum glucose and insulin concentrations were used to calculate the homeostasis model assessment-insulin resistance (HOMA-IR) and basic insulin secretion function index (HOMA-B), which were calculated by the following equations:

$$\begin{array}{c}\mathrm{HOMA} \mbox{-} \mathrm{IR}:\mathrm{FSG}(\mathrm{mg}/\mathrm{dL})\times {\rm{FSI}}(\mu \mathrm{IU}/\mathrm{mL})/\mathrm{405}{\rm{.}}\\ \mathrm{HOMA} \mbox{-} {\rm{\beta }}:20\times {\rm{FSI}}(\mu \mathrm{IU}/\mathrm{mL})/(\mathrm{FSG}(\mathrm{mmol}/L)-{\rm{3}}\mathrm{.5}){\rm{.}}\end{array}$$

where FSI is fasting serum insulin concentration and FSG is fasting serum glucose.

### Oxidative stress analysis

In this study, ROS and malondialdehyde (MDA) were analyzed as indicators for oxidative stress using commercial kits.

### Transcriptomic analysis of hepatic gene expression

In this study, we utilized autophagy gene expression profiling by RT2 Profiler PCR Arrays-Mouse Autophagy (Qiagen). The specific genes included in the Real-Time PCR Array analysis are listed in Table 3.

**Table 3:** Autophagy pathway and genes listed by RT2 Profiler PCR Arrays-Mouse Autophagy.

Autophagy Machinery Components	
Autophagic Vacuole Formation:	Atg4b, Atg4c, Atg9a, Gabarap, Gabarapl1, Gabarapl2, Wipi1, Becn1, Ulk1, Map1lc3
Ubiquitination and phagophore expansion:	Atg12, Atg16l1, Atg16l2, Atg3, Atg5, Atg7, Hdac6, Map1lc3a
Vacuole targeting and autophagosome-lysosome Linkage	Lamp1, Npc1, Gabarap, Atg4b, Atg4c
Chaperone-mediated autophagy	Hsp90aa1, Hspa8
**Regulation of Autophagy**	
Co-Regulators of autophagy and apoptosis	Dram1, Akt1, App, Atg12, Atg5, Bad, Bak1, Bax, Bcl2, Bcl2l1 (BclXL), Becn1,Bid, Bnip3, Casp3, Casp8 (FLICE), Cdkn1b (p27Kip1), Cdkn2a (p16INK4a), Cln3, Ctsb, Cxcr4, Dapk1, Eif2ak3, Fadd, Fas(Tnfrsf6), Hdac1, Htt, Ifng, Igf1, Ins2, Mapk8 (JNK1), Mtor, Nfkb1, Pik3cg, Prkaa1 (Ampk), Pten, Snca, Sqstm1, Tgfb1, Tgm2,Tnf, Tnfsf10 (Trail), Trp53 (p53).
Co-Regulators of autophagy and the cell cycle	Bax, Cdkn1b (p27Kip1), Cdkn2a (p16INK4a), Ifng, Pten, Rb1, Tgfb1, Trp53 (p53).
Autophagy induction by intracellular pathogens	Eif2ak3, Ifng, Lamp1.
Autophagy in response to other intracellular signals	Ctsd, Ctss, Dram2 (Tmem77), Eif4g1, Esr1 (Erα), Gaa, Hgs, Mapk14(p38alpha), Pik3c3 (Vps34), Pik3r4, Rps6kb1, Tmem74, Ulk2, Uvrag.

### Liver sampling and extraction of total cellular RNA

A U-shaped incision was made in the abdominal cavities of the overnight fasting animals killed after the end of the experiment (20 weeks) to dissect their livers. After that, the liver tissue samples were washed with normal saline and kept at −80 °C until PCR processing.

RNeasy Plus Mini Kit was used to prepare approximately 30 mg of each sample to which 600 μl of RLT buffer was added, followed by centrifugation at maximum speed for 3 minutes to collect the supernatant. The solution was homogenized, poured in a gDNA Eliminator spin column in a 2-ml collection tube, and centrifuged at high-speed 8000 × g (≥10,000 rpm) for 30 seconds. The obtained solution was mixed by 600 μl of 70% ethanol, followed by pipetting up and down. Next, 700 μl of the mixture was poured into the RNeasy spin column and centrifuged at ≥8000 × g for 15 seconds, which subsequently was mixed by 700 μl of RW1 buffer and centrifuged at ≥8000 × g for 15 seconds. Afterward, 500 μl of the RPE buffer was transferred into the RNeasy spin column and centrifuged at ≥8000 × g for 15 seconds and again for 2 minutes. The RNeasy spin column was delivered into the collection tube, about 30–50 μl of RNase-free water was added, and finally centrifuged at ≥8000 × g speed for 1 minute. The precipitated phase contained RNA, which was kept until next testing.

### Production of cDNA

All samples were tested for RNA concentration to compute the required RNA volume using spectrophotometer (Nano Drop 1000, Thermo Scientific, Pittsburgh, PA) at the wavelengths of 260 and 280 nm. Then, the cDNA was synthesized by RT2 First Strand Kit. Therefore, the determined amount of RNA was added to GE buffer and RNase-free water with final volume of 10 μl to prepare the genomic DNA elimination mix, followed by incubation at a temperature of 42 °C for 5 minutes and then immediately in ice for one minute.

The kit protocol was used to prepare the reverse-transcription mix containing 5 × Buffer BC3, control P2, RE3 Reverse Transcriptase Mix and RNase-free water. Next, 10 μl of reverse-transcription mix and genomic DNA elimination mix were distributed into each tube, pipetted, and incubated at 42 °C for 15 minutes and again at 95 °C for 5 minutes and then immediately placed in ice to stop the reaction. Thereafter, 91 μl of RNase-free water was added to the mixture, pipetted for real-time PCR, and placed at −15 to −30 °C.

### The Real-Time PCR for RT2 Profiler PCR Arrays

After centrifuging RT2 SYBR Green Mastermix (for 10–15 s), PCR components mix was prepared using the values required for RT2 SYBR Green Mastermix, cDNA synthesis reaction and RNase-free water according to the kit protocol.

RT2 SYBR Green Mastermix was centrifuged for 10–15 seconds, which combined with cDNA synthesis components and RNase-free water were used for PCR components mix preparation based on the kit protocol. PCR mix (25 μl) was transferred into each well of the plate, left at ambient temperature between 15 and 25 °C for a minute, and centrifuged at 1000 g to remove the appeared bubbles. The kit guidelines (Roche LightCycler 480) were followed to set the real-time cycler program. The real-time cycler software was applied to calculate threshold cycle (C_T_). The attained data were analyzed using the 2^−^ΔΔCT method, indicating fold-change as fold upregulation (>1) and fold downregulation (<1).

The housekeeping genes in the present study included Actin, Beta (Actb), Beta-2 microglobulin (B2m), Glyceraldehyde-3-phosphate dehydrogenase (Gapdh), Heat shock protein 90 alpha (cytosolic), and class B member 1 (Hsp90ab1).

The expression levels of gene of interest (GOI) and HKG genes were divided for normalization of GOI to HKG.

$$\frac{{2}^{-{\rm{CT}}({\rm{GOI}})}}{{2}^{-{\rm{CT}}({\rm{HKG}})}}={2}^{-[{\rm{CT}}({\rm{GOI}})-{\rm{CT}}({\rm{HKG}})]}={2}^{-{\rm{\Delta }}\text{CT}}$$

Then, the normalized GOI expression in the experimental sample was divided by the normalized GOI expression in the control to calculate the fold change in gene expression:

$$\frac{{2}^{-{\rm{\Delta }}\text{CT}({\rm{expt}})}}{{2}^{-{\rm{\Delta }}\text{CT}({\rm{ctrl}})}}={2}^{-{\rm{\Delta }}{\rm{\Delta }}\text{CT}}$$

where ΔΔCT is equal to ΔCT (expt) − ΔCT (ctrl).

The following equation shows the complete calculation:

$$\frac{\frac{{2}^{-{\rm{\Delta }}\mathrm{CT}({\rm{GOI}})}\,\mathrm{expt}}{{2}^{-{\rm{\Delta }}\mathrm{CT}({\rm{HKG}})}{\rm{expt}}}}{\frac{{2}^{-{\rm{\Delta }}\mathrm{CT}({\rm{GOI}})}{\rm{ctrl}}}{{2}^{-{\rm{\Delta }}\mathrm{CT}({\rm{HKG}})}{\rm{ctrl}}}}=\frac{{2}^{-[{\rm{CT}}({\rm{GOI}})-{\rm{\Delta }}\mathrm{CT}({\rm{HKG}})]}{\rm{expt}}}{{2}^{-[{\rm{CT}}({\rm{GOI}})-{\rm{\Delta }}\mathrm{CT}({\rm{HKG}})]}{\rm{ctrl}}}=\,\frac{{2}^{-{\rm{\Delta }}\mathrm{CT}\mathrm{expt}}}{{2}^{-{\rm{\Delta }}\mathrm{CT}\mathrm{ctrl}}}={2}^{-{\rm{\Delta }}{\rm{\Delta }}\mathrm{CT}}$$

### Statistical analyses

Data were presented as means ± SE. All the results were analyzed using Graph Pad Prism (version 7.03). Statistical significance was determined using the one-way analysis of variance with the Tukey post-hoc test and non-parametric Kruskal-Wallis test. Statistical significance was set at p < 0.05.

### Gene Symbol Description

Akt1: Thymoma viral proto-oncogene 1, Ambra1: Autophagy/beclin 1 regulator 1, App: Amyloid beta (A4) precursor protein, Atg12: Autophagy-related 12 (yeast), Atg16l1: Autophagy-related 16-like 1 (yeast), Atg16l2: Autophagy related 16 like 2 (S. cerevisiae), Atg3: Autophagy-related 3 (yeast), Atg4b: Autophagy-related 4B (yeast), Atg4c: Autophagy-related 4 C (yeast), Atg5: Autophagy-related 5 (yeast), Atg7: Autophagy-related 7 (yeast), Atg9a: Autophagy-related 9 A (yeast), Atg9b: ATG9 autophagy related 9 homolog B (S. cerevisiae), Bad: BCL2-associated agonist of cell death, Bak1: BCL2-antagonist/killer 1, Bax: Bcl2-associated X protein, Bcl2: B-cell leukemia/lymphoma 2, Bcl2l1: Bcl2-like 1,Becn1: Beclin 1, autophagy related, Bid: BH3 interacting domain death agonist, Bnip3: B12 Mm.378890 NM_009760 Bnip3, Casp3: Caspase 3, Casp8: Caspase 8, Cdkn1b: Cyclin-dependent kinase inhibitor 1B, Cdkn2a: Cyclin-dependent kinase inhibitor 2A, Cln3: Ceroid lipofuscinosis, neuronal 3, juvenile (Batten, Spielmeyer-Vogt disease), Ctsb: Cathepsin B, Ctsd: Cathepsin D, Ctss: Cathepsin S, Cxcr4: Chemokine (C-X-C motif) receptor 4, Dapk1: Death associated protein kinase 1, Dram1: DNA-damage regulated autophagy modulator 1, Dram2: VDNA-damage regulated autophagy modulator 2, Eif2ak3: Eukaryotic translation initiation factor 2 alpha kinase 3, Eif4g1: Eukaryotic translation initiation factor 4, gamma 1, Esr1: Estrogen receptor 1 (alpha), Fadd: Fas (TNFRSF6)-associated via death domain, Fas: Fas (TNF receptor superfamily member 6), Gaa: Glucosidase, alpha, acid, Gabarap: Gamma-aminobutyric acid receptor associated protein, Gabarapl1: Gamma-aminobutyric acid (GABA) A receptor-associated protein-like 1, Gabarapl2: Gamma-aminobutyric acid (GABA) A receptor-associated protein-like 2, Hdac1: Histone deacetylase 1, Hdac6: Histone deacetylase 6, Hgs: HGF-regulated tyrosine kinase substrate, Hsp90aa1: Heat shock protein 90, alpha (cytosolic), class A member 1, Hspa8: Heat shock protein 8, Htt: Huntingtin, Ifng: Interferon gamma, Igf1: Insulin-like growth factor 1, Ins2: Insulin II, Irgm1: Immunity-related GTPase family M member 1, Lamp1: Lysosomal-associated membrane protein 1, Map1lc3a: Microtubule-associated protein 1 light chain 3 alpha, Mapk14: Mitogen-activated protein kinase 14, Mapk8: Mitogen-activated protein kinase 8, Mtor: Mechanistic target of rapamycin (serine/threonine kinase), Nfkb1: Nuclear factor of kappa light polypeptide gene enhancer in B-cells 1, p105, Npc1: Niemann Pick type C1, Pik3c3: Phosphoinositide-3-kinase, class 3, Pik3cg: Phosphoinositide-3-kinase, catalytic, gamma polypeptide, Pik3r4: Phosphatidylinositol 3 kinase, regulatory subunit, polypeptide 4, p150, Prkaa1: Protein kinase, AMP-activated, alpha 1 catalytic subunit, Pten: Phosphatase and tensin homolog, Rab24: RAB24, member RAS oncogene family, Rb1: Rb1 Retinoblastoma 1, Rgs19: Regulator of G-protein signaling 19, Rps6kb1: Ribosomal protein S6 kinase, polypeptide 1, Snca: Synuclein, alpha, Sqstm1: Sequestosome 1, Tgfb1: Transforming growth factor, beta 1, Tgm2: Transglutaminase 2, C polypeptide, Tmem74: Transmembrane protein 74, Tnf: Tumor necrosis factor, Tnfsf10: Tumor necrosis factor (ligand) superfamily, member 10, Trp53: Transformation related protein 53, Ulk1: Unc-51 like kinase 1 (C. elegans), Uvrag: UV radiation resistance associated gene,Wipi1: WD repeat domain, phosphoinositide interacting 1.
